# Association between subclinical coronary artery atherosclerosis and oral health—a study on a Swedish population

**DOI:** 10.1038/s41405-026-00406-3

**Published:** 2026-02-17

**Authors:** Jessica Berglundh Gottlieb, Göran Bergström, Cristiano Tomasi, Tord Berglundh, Jan Derks

**Affiliations:** 1 Department of Periodontology, Institute of Odontology, Sahlgrenska Academy, University of Gothenburg, Gothenburg, Sweden; 2 Department of Molecular and Clinical Medicine, Institute of Medicine, Sahlgrenska Academy, University of Gothenburg, Gothenburg, Sweden

**Keywords:** Periodontitis, Periodontitis

## Abstract

**Introduction:**

Oral health has been linked to cardiovascular disease (CVD), but its relationship to subclinical coronary artery atherosclerosis (SCAA) remains unclear. Using coronary computed tomography angiography (CCTA), we explored this association in an asymptomatic population.

**Material and method:**

A total of 410 non-smokers (193 women, mean age: 64.6 years), comprising 204 individuals with SCAA and 206 without (non-SCAA), were assessed through clinical and radiographic oral evaluations. Self-reported oral symptoms were scored by questionnaire. We used sex-stratified regression analysis and compared model performance with and without the addition of data on oral health through area under the curve (AUC). The reference model included age and history of smoking.

**Results:**

Individuals with SCAA had more missing teeth, higher Decayed and Filled Teeth scores and greater prevalence of peri-apical lesions and marginal bone loss >33%. Missing teeth was an independent risk indicator for SCAA (OR 1.15 95%CI 1.04–1.27). Model performance improved with the addition of oral status and self-reported oral symptoms, most prominently in women (AUC 0.67 vs. 0.78, *p* = 0.010). Decision curve analyses confirmed a consistent net benefit when data on oral health were considered.

**Conclusions:**

The findings suggest that subclinical coronary artery atherosclerosis is associated with oral health. Oral health-related data may improve screening for risk of coronary events, especially in women.

## Introduction

Cardiovascular disease (CVD) is the most common cause of death on a global perspective, accounting for 9 million deaths annually [1]. Myocardial infarction (MI) is a frequent and fatal CVD event [2] and results from ruptures of atherosclerotic plaque in the coronary arteries [3]. Chronic inflammation contributes to atherosclerosis and CVD [4], where oral conditions, such as periodontitis and endodontic inflammatory disease, represent potential contributors [5, 6].

The association between CVD and oral health has been studied through two general approaches. Firstly, studies have targeted the occurrence of severe CVD-events (e.g., MI), demonstrating a link with tooth loss [5, 7, 8], periodontal disease [9, 10], and endodontic inflammatory disease [11, 12]. Furthermore, untreated caries, peri-apical lesions and high Decayed, Missing and Filled Teeth scores have been linked to an increased risk for MI [7]. In a second strategy, studies addressed CVD-risk profiles in asymptomatic populations, for which the majority utilized assessments of carotid intima-media thickness (c-IMT). It was demonstrated that periodontitis and tooth loss were overrepresented among patient groups at elevated, c-IMT-assessed risk for CVD [13–17]. The relevance of c-IMT in the prediction of future MI events, however, has been questioned due to poor accuracy [18–20]. Novel imaging techniques, such as coronary artery calcium (CAC) imaging and coronary computed tomography angiography (CCTA) detect subclinical coronary artery atherosclerosis (SCAA) with high precision, thereby improving prediction of cardiac events in population studies [21–25]. Recently, a large, cross-sectional evaluation performed CCTA in an asymptomatic, representative middle-aged Swedish population sample. SCAA was a common finding (42%) and overrepresented in men [25].

In light of the limited evidence on the association between oral health and subclinical CVD, it is evident that more research is required. To fill this gap, we evaluated an asymptomatic population sample, aiming to disclose the relationship between oral health and CCTA-based risk for cardiac events.

## Material and methods

### Data source and study population

The protocol of the present case-control study was approved by the Swedish Ethical Review Authority (Registration number 2021-03428). Reporting follows STROBE [26] and SAGER guidelines [27].

Study participants were selected from a pool of 6500 individuals from the Swedish CArdio and Pulmonary BioImage Study (SCAPIS) center in Gothenburg [25, 28]. Eligible subjects had completed the SCAPIS examination procedures during the period 2014–2018, including extensive questionnaires on self-reported medical data (i.e., medical health, smoking habits, medications), anthropometric assessments (i.e., waist circumference, body mass index (BMI)), and physiological and radiographic examinations. Presence of subclinical coronary artery atherosclerosis (SCAA) was evaluated through coronary computed tomography angiography (CCTA). The segment involvement score (SIS) [29]; was applied to categorize individuals as either negative (SIS = 0; non-SCAA) or positive (SIS ≥ 3; SCAA). Additional inclusion criteria for the present analyses, were no history of myocardial infarction (MI), no previous coronary intervention (i.e., percutaneous coronary intervention or coronary artery bypass graft) prior to the participation in SCAPIS. Due to possible confounding effect [30] and sample size constraints, individuals with a known diagnosis of diabetes and current tobacco smokers were excluded (former smokers allowed).

Invitations were sent by mail, and subjects were subsequently contacted by telephone. The stratified inclusion process was overseen by one study coordinator (ACE) and concluded once 200 cases with SCAA and 200 without SCAA were enrolled. Out of 647 invited subjects, 425 accepted and were subsequently examined (response rate: 65.7%). All examinations were carried out during the same day. Fifteen subjects were excluded after examination (Supplementary material Fig. S1, Table S1).

### Self-reported oral symptoms

Following an initial interview addressing general and oral health status and oral hygiene habits, the participants completed a seven-item questionnaire on oral symptoms [31] (Table 1). Responses to the unweighted questionnaire items were dichotomized: yes or no (the response “don’t know” was treated as missing data). Oral symptoms were summarized by number of positive answers: yes (0, 1, 2 or ≥3).

**Table 1:** Oral status and self-reported symptoms.

	All (*n* = 410)					Men (*n* = 217)					Women (n = 193)				
	SCAA (*n* = 204)		Non-SCAA (*n* = 206)			SCAA (*n* = 152)		Non-SCAA (*n* = 65)			SCAA (*n* = 52)		Non-SCAA (n = 141)		
	n/Mean	%/(SD)	n/Mean	%/(SD)	SMD	n/Mean	%/(SD)	n/Mean	%/(SD)	SMD	n/Mean	%/(SD)	n/Mean	%/(SD)	SMD
Number of teeth	25.5	(3.1)	26.4	(2.0)	-0.33	25.7	(3.0)	26.3	(2.0)	-0.24	24.9	(3.3)	26.4	(1.9)	-0.54
Number of missing teeth	2.1	(3.1)	1.1	(1.8)	0.37	1.9	(3.0)	1.3	(1.9)	0.23	2.5	(3.2)	1.1	(1.7)	0.57
Number of teeth with untreated decay	0.9	(1.4)	0.6	(0.9)	0.24	0.9	(1.5)	0.7	(0.9)	0.21	0.8	(1.2)	0.6	(1.0)	0.18
DFT	14.1	(5.1)	12.9	(4.7)	0.25	13.7	(5.1)	12.9	(4.9)	0.15	15.5	(4.7)	12.9	(4.6)	0.54
Number of teeth with coronal restorations	13.6	(5.1)	12.5	(4.7)	0.23	13.2	(5.2)	12.5	(4.9)	0.14	14.7	(5.0)	12.4	(4.7)	0.46
Number of teeth with root fillings	1.2	(1.6)	1.2	(1.6)	0.05	1.1	(1.4)	1.4	(1.6)	-0.18	1.5	(2.2)	1.0	(1.5)	0.26
Number of teeth with peri-apical radiolucencies	0.9	(1.3)	0.7	(1.0)	0.17	1.0	(1.3)	0.9	(1.2)	0.04	0.9	(1.3)	0.7	(1.0)	0.19
Number of teeth with bone loss ( > 33%)	0.9	(1.7)	0.6	(1.6)	0.16	0.7	(1.6)	0.7	(2.3)	-0.03	1.4	(1.9)	0.5	(1.3)	0.58
Number of subjects presenting with ≥1 tooth with PD ≥ 6 mm	72	35.3%	53	25.7%	0.21	51	33.6%	19	29.2%	0.09	21	40.4%	34	24.1%	0.35
Periodontal status															
Healthy	0	0	0	0	0.31	0	0	0	0	0.31	0	0.0%	0	0.0%	0.55
Localized gingivitis	2	1%	3	1.5%		2	1.3%	2	3.0%		0	0.0%	1	0.7%	
Generalized gingivitis	6	2.9%	5	2.4%		6	3.9%	2	3.0%		0	0.0%	3	2.1%	
Stage I	46	22.6%	70	34.0%		34	22.4%	23	35.4%		12	23.1%	47	33.3%	
Stage II	33	16.2%	34	16.5%		23	15.1%	7	10.8%		10	19.2%	27	19.2%	
Stage III	94	46.1%	83	40.3%		74	48.7%	26	40.0%		20	38.5%	57	40.4%	
Stage IV^a^	23	11.2%	11	5.3%		13	8.6%	5	7.8%		10	19.2%	6	4.3%	
Gingival health on a reduced periodontium ( < 10% bleeding sites + no PD < 5 mm)	8/196		4/198		0.12	6/144		0/61		0.29	2/52		4/137		0.05
Gingival inflammation on a reduced periodontium ( ≥ 10% bleeding sites + no PD < 4 mm)	9/196		8/198		0.03	5/144		1/61		0.12	4/52		7/137		0.11
*Have you during the last year experienced:*															
Swollen gums (yes)	45	22.6%	47	23.9%	0.03	29	19.6%	14	23.0%	0.08	16	31.4%	33	24.3%	0.16
Sore gums (yes)	89	44.5%	116	56.9%	0.25	63	42.3%	43	66.2%	0.49	26	51.0%	73	52.5%	0.03
Receding gums (yes)	47	26.4%	78	42.4%	0.34	31	23.0%	20	33.9%	0.24	16	37.2%	58	46.4%	0.19
Loose teeth (yes)	11	5.5%	8	3.9%	0.08	8	5.4%	3	4.6%	0.03	3	6.0%	5	3.6%	0.11
Drifting teeth (yes)	16	8.1%	15	7.7%	0.01	10	6.8%	5	7.8%	0.04	6	12.2%	10	7.7%	0.15
Bad breath (yes)	35	19.9%	40	22.3%	0.06	25	18.9%	14	24.6%	0.14	10	22.7%	26	21.3%	0.03
Toothache (yes)	31	15.3%	42	20.6%	0.14	23	15.3%	11	17.2%	0.05	8	15.4%	31	22.1%	0.17
Number of symptoms															
0	76	37.3%	44	21.4%	0.43	61	40.1%	12	18.5%	0.53	15	28.8%	32	22.7%	0.22
1	94	46.1%	107	51.9%		69	45.4%	37	56.9%		25	48.1%	70	49.6%	
2	23	11.3%	47	22.8%		14	9.2%	13	20.0%		9	17.3%	34	24.1%	
>3	11	5.4%	8	3.9%		8	5.3%	3	4.6%		3	5.8%	5	3.5%	

An extended Table 1 is found in the Supplementary Material (Table S3).

*DFT* Decayed and Filled Teeth, *PD* Probing Depth, *SCAA* Subclinical Coronary Artery Atherosclerosis, *SD* Standard Deviation, *SMD* Standardized Mean Difference.

^a^Information on reasons for tooth loss was not available. Loss of > 4 teeth was used a criterium for stage IV.

### Clinical examinations

Examinations were performed at the Specialist Clinic of Periodontics in Gothenburg (Public Dental Services, Region Västra Götaland, Sweden) by three calibrated dentists (JBG, SH, ES), blinded to grouping. The clinical examination included assessments of number of missing teeth (disregarding aplasia and orthodontic extractions), caries lesions, dental implants and restorations. Probing depth (PD), bleeding on probing (BoP) and gingival recession (GR) were registered at mesial, buccal, distal and lingual aspects of each tooth/implant by means of a periodontal probe (Hu-Friedy Inc., Leimen, Germany). Clinical attachment levels were calculated (PD + GR). Furcation involvement and tooth mobility were also scored. Periodontitis was classified according to the 2017 World Workshop on the Classification of Periodontal and Peri-Implant Diseases and Conditions [32, 33]. Third molars were not considered.

### Radiographic analysis

Digital panoramic radiographs of each participant were obtained at the Specialist Clinic of Oral Radiology (Public Dental Services, Region Västra Götaland, Sweden) using ProMax 3D (Planmeca, Helsinki, Finland). Assessments were performed (PACS; Sectra IDS7 (Sectra AB, Linköping, Sweden)) by three calibrated observers (JBG, LA, DS), blinded to grouping. We scored number of remaining teeth and noted presence of caries, root fillings and restorations. Peri-apical radiolucencies were categorized according to Sebring et al. [12].

**Fig. 1:**
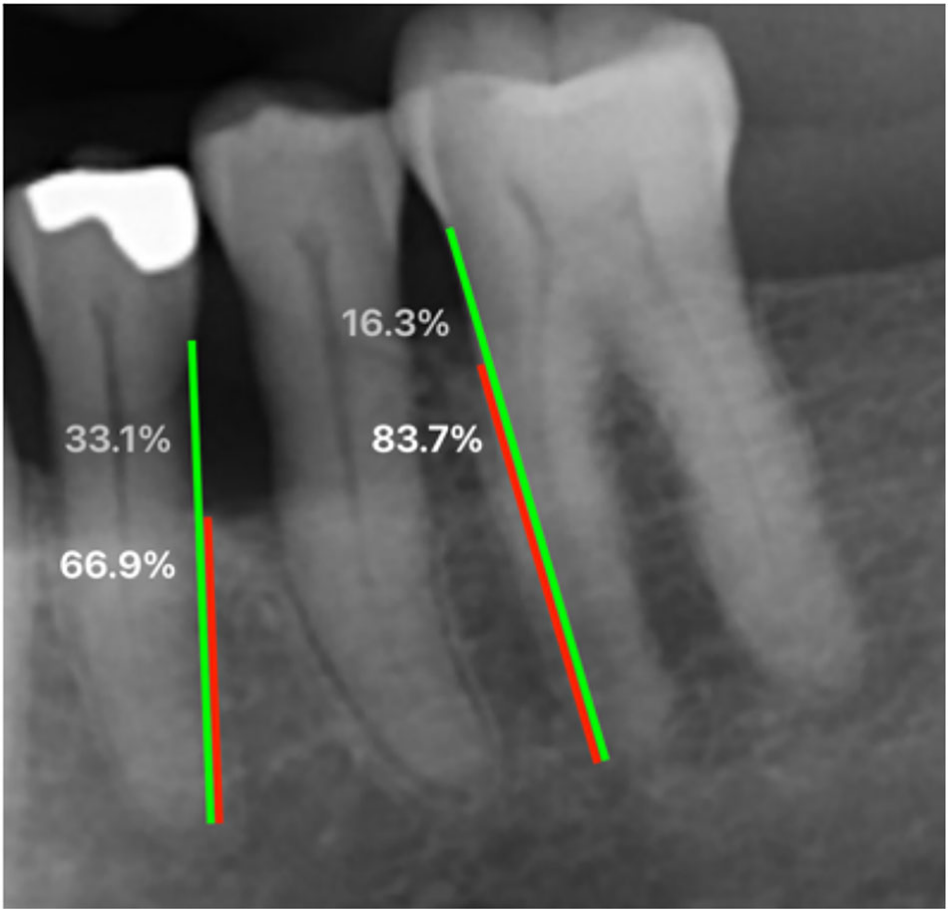
Radiographic measurements. Radiographic assessments were performed by measuring the distance from the marginal alveolar bone to the tooth apex (red line) and from the cementoenamel junction to the tooth apex (green line). The proportions of remaining bone and bone loss are depicted in white and gray, respectively. Bone loss exceeding 33% (as presented at the first premolar in this figure) were used as a threshold.

The distance between the cementoenamel junction and tooth apex (root length), and the distance between the marginal bone crest and tooth apex (bone height) were measured at mesial and distal sites at each tooth (Fig. 1). Marginal bone level (MBL) was expressed as percentage of root length. The distal sites of second molars were excluded from this part of the analyses. Double measurement of MBL on 20 radiographic images revealed an inter-examiner agreement (intra-class correlation) of 0.90 (95%CI 0.89–0.92).

### Statistical analysis

Based on a priori calculations, a sample size of 400 participants was found to be sufficient to detect a 15% difference in periodontitis prevalence between groups (alpha = 0.05, power 80%).

Continuous parameters were described as mean values (± standard deviation) and categorical variables were expressed as proportions. Standardized mean differences between SCAA and non-SCAA individuals were calculated for continuous and categorical characteristics using the *stddiff* command [34].

To evaluate the association between oral health and SCAA, we used logistic regression analyses (dependent parameter: SCAA). A first model was limited to the independent parameters age and history of smoking (model 1). In a second step, parameters on oral status were added (model 2; i.e., number of missing teeth, number of Decayed and Filled Teeth (DFT), number of teeth with marginal bone loss (MBL ≥ 33%), and number of teeth with peri-apical radiolucencies). Thirdly, the model was extended to also include information on self-reported oral symptoms (model 3). Model performance was described by Area under the Receiver Operating Characteristic curve (AUC) and compared using the *rocgold* command (Chi^2^-testing with Bonferroni correction). All analyses were repeated within strata of sex. An extended model 1 was built as reference including additional personal/medical information. We also evaluated the impact of individual symptoms (questionnaire data) in sensitivity analyses.

Decision curve analysis (DCA) was performed to assess the clinical utility of the models [35]. For the DCA, overall prevalence of SCAA was set to 42% and adapted by sex [25]. Predicted probabilities were internally validated using bootstrap resampling (1,000 repetitions). Model coefficients were globally shrunk using the optimism-corrected calibration slope, followed by intercept recalibration.

All estimates were accompanied by 95% confidence intervals (CI). Statistical analyses were performed using Stata 19 (StataCorp, College Station, TX, US).

### Ethics approval

This study involves human participants and was approved by the Swedish Ethical Review Authority (Registration number 2021-03428). Participants gave informed consent to participate in the study before taking part.

## Results

### Study sample

In total, 410 subjects (SCAA: *n* = 204; non-SCAA: *n* = 206) were included in the analysis. The mean age at the time of dental examination was 64.6 ± 4.6 years. The mean time between SCAPIS participation and dental examination was 6.6 ± 1.2 years. In subjects with SCAA, the mean SIS was 4.8 ± 1.8, with values ranging from 3 to 10. Men were overrepresented in the SCAA-group, as was a history of smoking (Table 2 and S2).

**Table 2:** Descriptive characteristics of study participants.

	All				Men				Women			
	SCAA (*n* = 204)		Non-SCAA (*n* = 206)		SCAA (*n* = 152)		Non-SCAA (*n* = 65)		SCAA (*n* = 52)		Non-SCAA (n = 141)	
	n/Mean	%/(SD)	n/Mean	%/(SD)	n/Mean	%/(SD)	n/Mean	%/(SD)	n/Mean	%/(SD)	n/Mean	%/(SD)
Age	65.8	(4.4)	63.5	(4.4)	65.7	(4.4)	62.8	(3.9)	66.1	(4.5)	63.8	(4.6)
Time from SCAPIS to dental examination	6.6	(1.2)	6.6	(1.2)	6.6	(1.2)	6.5	(1.2)	6.6	(1.3)	6.6	(1.1)
SIS (Segment Involvement Score)^a^	4.8	(1.8)	0.0	(0.0)	4.9	(1.8)	0.0	(0.0)	4.3	(1.5)	0.0	(0.0)
Highest completed level of education^a^												
High school	91	44.6%	79	38.3%	66	43.4%	23	35.4%	25	48.1%	56	39.7%
University	99	48.5%	111	53.9%	75	49.3%	37	56.9%	24	46.2%	74	52.5%
History of smoking^a^												
Never	96	47.1%	127	61.7%	75	49.3%	44	67.7%	21	40.4%	83	58.9%
Previous	108	52.9%	79	38.3%	77	50.7%	21	32.3%	31	59.6%	58	41.1%
*Pack-years*	13.8	(11.7)	9.6	(8.0)	13.8	(12.6)	8.8	(7.0)	13.8	(9.3)	9.9	(8.4)
<10	46	42.6%	46	58.2%	34	44.2%	12	57.1%	12	38.7%	34	58.6%
10-24	46	42.6%	29	36.7%	32	41.6%	8	38.1%	14	45.2%	21	36.2%
25-39	12	11.1%	4	5.1%	7	9.1%	1	4.8%	5	16.1%	3	5.2%
≥40	4	3.7%	0	0.0%	4	5.2%	0	0.0%	0	0.0%	0	0.0%
BMI (Body Mass Index kg/m2)^a^	26.5	(3.5)	25.5	(4.2)	26.8	(3.5)	26.1	(3.2)	25.8	(3.7)	25.3	(4.5)
Underweight <18.5	0	0.0%	1	0.5%	0	0.0%	0	0.0%	0	0.0%	1	0.7%
Normal 18.5–24.9	75	36.8%	110	53.4%	52	34.2%	29	44.6%	23	44.2%	81	57.4%
Overweight 25–29.9	96	47.1%	70	34.0%	74	48.7%	28	43.1%	22	42.3%	42	29.8%
Obesity ≥30	33	16.2%	25	12.1%	26	17.1%	8	12.3%	7	13.5%	17	12.1%
Waist (cm)^a^	95.7	(11.2)	89.0	(11.7)	98.3	(10.4)	96.0	(9.4)	88.2	(9.9)	85.7	(11.3)
Dental visits per year (self-reported)												
Never, only emergency	13	6.4%	9	4.4%	11	7.2%	5	7.7%	2	3.8%	4	2.8%
<1/year	45	22.1%	52	25.2%	39	25.7%	18	27.7%	6	11.5%	34	24.1%
1/year	90	44.1%	93	45.1%	64	42.1%	23	35.4%	26	50.0%	70	49.6%
2/year	56	27.5%	52	25.2%	38	25.0%	19	29.2%	18	34.6%	33	23.4%

An extended Table 2 is found in the Supplementary Material (Table S2).

^a^Information collected at the time of SCAPIS examination 2012–2019.

*SCAA* Subclinical Coronary Artery Atherosclerosis, *SD* Standard Deviation.

### Oral health and SCAA

**Fig. 2:**
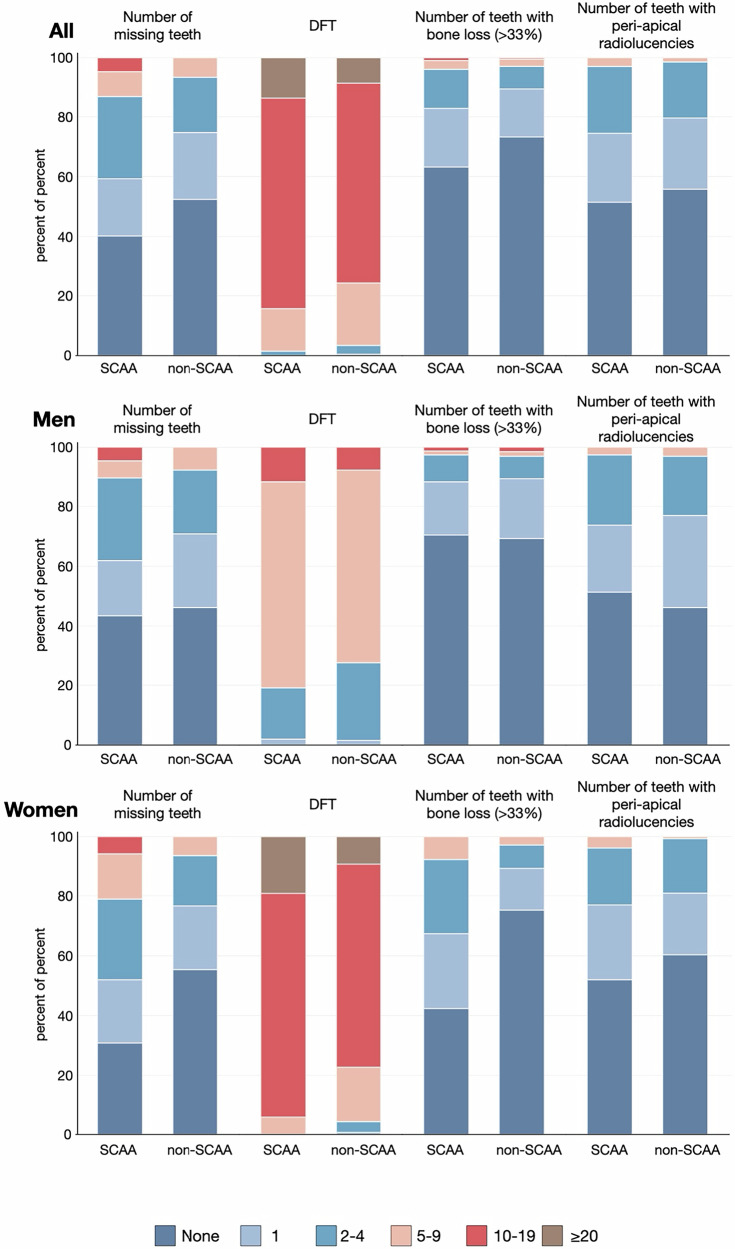
Oral status in SCAA and non-SCAA groups, stratified by gender. Categories assessed include number of missing teeth, number of decayed and filled teeth (DFT), number of teeth with marginal bone loss ( > 33%) and number of teeth with peri-apical radiolucencies.

An overview of our findings is provided in Tables 1, S3, S4, S5, S6 and Figs. 2, S2. All participants were dentate. Individuals with SCAA presented higher mean values than non-SCAA in terms of number of missing teeth, DFT, number of teeth with peri-apical radiolucencies and number of teeth with marginal bone loss ( ≥ 33%). Out of the 410 participants, 290 reported ≥1 oral symptom, and a total of 19 suffered from ≥3 symptoms. Non-SCAA individuals reported more symptoms than SCAA.

Group-related differences in clinical and radiographic parameters were primarily evident among women, favoring non-SCAA. Women with SCAA presented with higher DFT scores as well as higher numbers of missing teeth and teeth affected by severe bone loss than men and women without SCAA (Fig. 2).

**Fig. 3:**
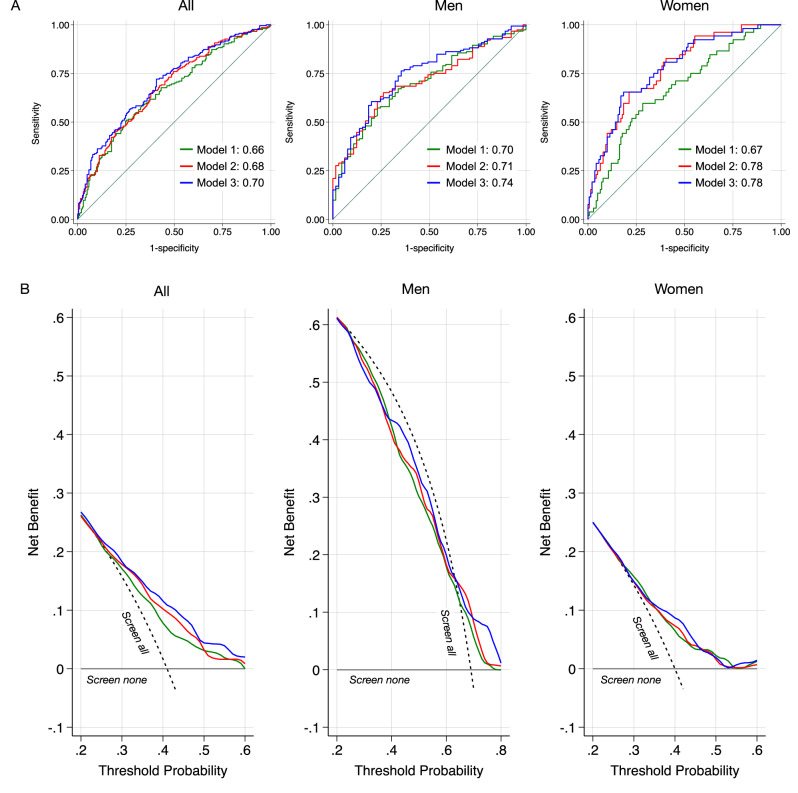
Comparison of the three prognostic models (green = model 1, red = model 2, blue = model 3) in the overall study sample, in men, and in women. See Table 3 for details on the regression analyses. **A** Area under the receiver operating curve. Model 3 achieved the highest discrimination. The inclusion of oral health parameters improved model performance in women. **B** Decision curve analysis (internally validated). Net benefit of each model is shown across threshold probabilities at which a patient/clinician would opt for screening/intervention. The “screen all” strategy assumes that all individuals undergo screening, whereas “screen none” assumes the opposite. A model is considered to provide clinical benefit (curves are positioned to the right of the “screen all” line), if the number of true positives (correctly diagnosed) is increased while decreasing false positives over a meaningful range of thresholds. Across the examined thresholds, Models 2 and 3 demonstrate higher net benefit than Model 1, particularly in women. **–** Model 1: SCAA explained by age, history of smoking. **–** Model 2: SCAA explained by Model 1 + number of missing teeth, DFT, number of teeth with peri-apical radiolucencies, number of teeth with marginal bone loss ( > 33%). **–** Model 3: SCAA explained by Model 2 + self-reported oral symptoms.

Number of missing teeth was an independent risk indicator for SCAA (OR 1.15 95%CI 1.04–1.27). Overall, model performance was mildly improved by the addition of items describing oral health (Table 3 and Figs. 3A, S3). While the addition of clinical and radiographic findings alone did not enhance the model (*p* = 0.242), the additional consideration of self-reported symptoms was beneficial (*p* = 0.039). The sex-stratified analysis revealed that information on oral health did not improve accuracy over the reference model in men. For women, oral health status did substantially improve discrimination, illustrated by an increase in AUC from 0.67 to 0.78 (*p* = 0.010). Self-reported data did not result in further improvement beyond model 2. DCA illustrated the net benefit of the inclusion of oral health information (model 3) over a wide range of probability thresholds (Fig. 3B). Model-based screening provided clearer separation in net benefit among men, whereas benefits were more modest and overlapping in women.

**Table 3:** Results of the overall and gender-stratified logistic regression analyses (dependent parameter: SCAA) presented as odds ratios and 95% confidence intervals.

	All (*n* = 410)			Men (*n* = 217)			Women (*n* = 193)		
*Independent parameters*	Model 1	Model 2	Model 3	Model 1	Model 2	Model 3	Model 1	Model 2	Model 3
**Age**	1.12 (1.07–1.17)**	1.10 (1.05–1.16)**	1.10 (1.04–1.16)**	1.16 (1.08–1.25)**	1.16 (1.08–1.26)**	1.17 (1.08–1.27)**	1.11 (1.03–1.20)**	1.04 (0.96–1.14)	1.04 (0.95–1.13)
**History of smoking** (reference: none)	1.64 (1.09–2.45)*	1.48 (0.98–2.25)	1.49 (0.97–2.27)	1.84 (0.97–3.47)	1.78 (0.92–3.45)	1.65 (0.84–3.26)	2.01 (1.04–3.90)*	1.88 (0.91–3.91)	1.93 (0.92–4.04)
**Number of missing teeth**		1.15 (1.04–1.27)**	1.14 (1.02–1.27)*		1.08 (0.93–1.26)	1.05 (0.89–1.23)		1.23 (1.04–1.47)*	1.25 (1.04–1.49)*
**DFT**		1.02 (0.97–1.07)	1.02 (0.97–1.07)		0.99 (0.93–1.07)	0.98 (0.91–1.06)		1.14 (1.04–1.25)**	1.14 (1.04–1.24)**
**Number of teeth with bone loss (>33%)**		1.02 (0.90–1.16)	1.02 (0.89–1.17)		0.95 (0.81–1.11)	0.93 (0.79–1.10)		1.37 (1.07–1.75)*	1.37 (1.07–1.76)*
**Number of teeth with peri-apical radiolucencies**		1.00 (0.83–1.21)	1.01(0.84–1.23)		0.94 (0.72–1.23)	0.95 (0.72–1.25)		0.87 (0.61–1.23)	0.87 (0.61–1.24)
**Self-reported oral symptoms** (reference: none)									
1			0.60 (0.37–0.98)*			0.46 (0.21–0.99)*			0.97 (0.40–2.34)
2			0.34 (0.18–0.67)**			0.26 (0.09–0.73)*			0.61 (0.19–1.92)
≥3			0.97 (0.33–2.84)			1.10 (0.22–5.55)			1.17 (0.19–7.32)
**AUC**	0.66 (0.61–0.71)	0.68 (0.63–0.73)	0.70 (0.65–0.75)	0.70 (0.63–0.77)	0.71 (0.64–0.78)	0.74 (0.67–0.81)	0.67 (0.59–0.76)	0.78 (0.70–0.85)	0.78 (0.71–0.85)
*Comparison of model performance*	reference	vs. Model 1 p = 0.242	vs. Model 1 p = 0.039	reference	vs. Model 1 p = 1.000	vs. Model 1 p = 0.221	reference	vs. Model 1 p = 0.010	vs. Model 1 p = 0.010

Model 1 includes only background parameters, while the extend models include clinical/radiographic information (Model 2) and self-reported symptoms (Model 3).

*AUC* Area under the Receiver Operating Characteristics curve, *DFT* Decayed and Filled Teeth, *SCAA* Subclinical Coronary Artery Atherosclerosis.

**p < .01,*p < .05 (Chi^2^-test corrected for Bonferroni).

## Discussion

This study is unique as it uses CCTA-verified subclinical coronary atherosclerosis (SCAA) in its analysis of the relationship between oral status and risk for cardiac events. The findings revealed that individuals with SCAA (assessed 6.6 years earlier) exhibited more missing teeth, higher DFT scores, and greater prevalence of peri-apical lesions and severe bone loss. The addition of information on oral status and self-reported oral symptoms improved discrimination substantially, particularly in women. Decision curve analysis further demonstrated that the incorporation of oral health data yielded consistent net benefit.

One novelty of this study lies in its focus on linking oral health to CVD risk profiles assessed in an asymptomatic population. Thereby, our strategy differed from the bulk of the existing evidence, considering patient groups already affected by serious cardiac events [9]. Despite the fundamentally different approach, the findings of higher proportions of tooth loss and periodontitis in patients with a first MI (myocardial infarction) shown by Rydén et al. [9] corroborate the data presented in this study.

A further novelty was the use of CCTA-verified SCAA to inform risk profiles. The use of CCTA distinguishes itself from previous evaluations in the field, which were typically limited to carotid assessments [13–17]. As carotid intima wall thickness (c-IMT) has been questioned as to its accuracy in predicting future cardiac events [18–20], the use of CCTA in the present dataset probably represents a strength. Nevertheless, earlier findings based on c-IMT are in line with our results, suggesting an overrepresentation of tooth loss and periodontitis in risk individuals [13–17].

Among the different discriminatory strategies tested for identifying individuals with SCAA, the model including information on both oral status and symptoms (Model 3) consistently showed the best discrimination and clinical utility. The net benefit illustrated by the decision curve analysis should be interpreted considering threshold probability in the given context. Our reference test (CCTA) is demanding in terms of resources and entails limitations in terms of access. This would correspond to higher threshold probabilities, the area in which the oral data-based models demonstrated benefits. This is particularly relevant as screening of oral status is easy, not time-consuming and minimally invasive. This is also reflected in our choice of covariates included in the models (i.e., age, history of smoking). The simplistic approach was to mimic routine care in a dental setting. It is important to note that the model including covariates corresponding to medical information, still outperformed the oral health-based model.

Model performance was better in women than in men. This finding is probably explained by the fact that the subgroup of women with SCAA showed the highest scores related to tooth loss, caries and periodontitis. Another study performed in a Swedish population evaluated the risk of a first MI by oral status and found a relevant association only in women [36], indirectly confirming our data. It is important to note that our dataset was strongly imbalanced in terms of sex distribution across groups, as men were highly over-represented in the SCAA-group. This may explain the difference in net-benefit between sexes and may have limited the validity of our sex-specific estimates.

The addition of self-reported oral symptoms improved the risk model performance in our dataset. Poor self-related oral health has been linked to cardiovascular risk derived from a SCORE 2 questionnaire [37]. Somewhat in line with our findings, the association could be demonstrated for women, only. It is important to note, that self-reported oral health may not necessarily reflect oral disease burden but rather illustrates disease awareness [38].

It must be noted that our findings of a stronger association between SCAA and oral health in women is not in agreement with several previous investigations. Multiple c-IMT-based studies reported contrary findings, i.e., the link was most prominent among men [15, 39, 40]. The reason for this inconsistency is not understood but may be related to the methodology of CVD risk assessments.

A limitation of this study is the fact that our dental assessment was performed approximately 6 years after the CCTA evaluation. It is unlikely, however, that pronounced changes in oral status in the present age category occurred during this time frame [41]. The SCAA-categorization was based on SIS = 0 versus SIS ≥ 3. By applying these distinct and separated thresholds, minor shifts in SIS during the 6-year period would not have affected the overall risk profile.

## Conclusion

The findings suggest that subclinical coronary artery atherosclerosis is associated with oral health. Oral health-related data may improve screening for risk of coronary events, especially in women.

## Supplementary information

Supplementary Material

## Data Availability

Data are available upon reasonable request. The de-identified participant data that underlie the results reported in this article, as well as the statistical code are available from the corresponding author upon reasonable request and upon a signed data access agreement.
