# Supplementary material for: Association between subclinical coronary artery atherosclerosis and oral health—a study on a Swedish population
Source: BDJ Open. 2026 Feb 17;12:18. doi: 10.1038/s41405-026-00406-3 (PMC12913598; doi:10.1038/s41405-026-00406-3)
Supplement: Supplementary file 1 — Supplementary Material [file 41405_2026_406_MOESM1_ESM.docx]

Supplementary material

#### Table S1. Response to invitation

|  | **No reply** | | **Declined** | | **Accepted** | | **Total** | |
| --- | --- | --- | --- | --- | --- | --- | --- | --- |
| **Sex** |  |  |  |  |  |  |  |  |
| Women | 71 | 47.7% | 33 | 45.2% | 202 | 47.5% | 306 | 47.3% |
| Men | 78 | 52.3% | 40 | 54.8% | 223 | 52.5% | 341 | 52.7% |
| **Group** |  |  |  |  |  |  |  |  |
| SCAA | 69 | 46.3% | 42 | 57.5% | 213 | 50.1% | 324 | 50.1% |
| non-SCAA | 80 | 53.7% | 31 | 42.5% | 212 | 49.9% | 323 | 49.9% |
| **Age** | 63.0 | (4.4) | 64.4 | (4.1) | 64.5 | (4.6) | 64.2 | (4.5) |
| *Data presented as means and standard deviations or frequencies and proportions.* | | | | | | | | |

Table S2. Descriptive characteristics of study participants

|  | **SCAA**  **(n=204)** | | **Non-SCAA**  **(n=206)** | |
| --- | --- | --- | --- | --- |
| **Snuff*** |  |  |  |  |
| None | 164 | 80.8% | 195 | 95.1% |
| Previous | 10 | 4.9% | 3 | 1.5% |
| Daily | 29 | 14.3% | 7 | 3.4% |
| **Event after SCAPIS** |  |  |  |  |
| None | 192 | 94.1% | 203 | 98.5% |
| MI | 11 | 5.4% | 0 | 0.0% |
| Stroke | 1 | 0.5% | 3 | 1.5% |
| **Heredity for MI*** | 19 | 9.3% | 16 | 7.8% |
| **Heredity for Stroke*** | 17 | 8.3% | 5 | 2.4% |
| ***Disease*** |  |  |  |  |
| Hypertension | 112 | 54.9% | 39 | 18.9% |
| Peripheral artery disease | 2 | 1.0% | 2 | 1.0% |
| Dyslipidemia | 79 | 38.7% | 17 | 8.3% |
| Obstructive lung disease | 5 | 2.5% | 2 | 1.0% |
| Kidney disease | 1 | 0.5% | 1 | 0.5% |
| Cancer | 4 | 2.0% | 7 | 3.4% |
| Depression | 13 | 6.4% | 13 | 6.3% |
| Rheumatic disease | 5 | 2.5% | 7 | 3.4% |
| Thyreoidea | 9 | 4.4% | 21 | 10.2% |
| Arythmia | 8 | 3.9% | 6 | 2.9% |
| ***Medication*** |  |  |  |  |
| Renin-Angiotensin inhibitors | 75 | 36.8% | 29 | 14.1% |
| Calcium antagonist | 34 | 16.7% | 10 | 4.9% |
| Aspirin | 37 | 18.1% | 7 | 3.4% |
| Beta-blockers | 25 | 12.3% | 11 | 5.3% |
| NOAK | 10 | 4.9% | 4 | 1.9% |
| Waran | 1 | 0.5% | 0 | 0.0% |
| Statins | 77 | 37.7% | 16 | 7.8% |
| Cortison | 7 | 3.4% | 5 | 2.4% |
| ***Physical activity**** |  |  |  |  |
| Low-intensity physical activity (min/day) | 339.8 | (83.3) | 351.2 | (79.3) |
| Moderate- intensity-physical activity (min/day) | 50.6 | (30.5) | 49.5 | (21.6) |
| Moderate- and vigorous-intensity physical activity (min/day) | 55.4 | (33.1) | 55.7 | (24.5) |
| Moderate- and vigorous-intensity physical activity (% wear time/day) | 6.4 | (3.9) | 6.4 | (2.9) |
| Sedentary (% wear time/day) | 54.8 | (10.1) | 53.4 | (10.0) |
| **Frequency having an alcoholic drink, last year*** |  |  |  |  |
| Never | 14 | 6.9% | 11 | 5.3% |
| Once/month or less | 25 | 12.3% | 27 | 13.1% |
| 2-4/month | 76 | 37.3% | 74 | 35.9% |
| 2-3/week | 70 | 34.3% | 81 | 39.3% |
| 4/week or more | 19 | 9.3% | 13 | 6.3% |
| **Information collected at the time of SCAPIS examination 2012-2019.*  *Data presented as means and standard deviations or frequencies and proportions.* | | | | |

Table S3: Extended table of oral status parameters.

|  | **All (n=410)** | | | | | **Men (n=217)** | | | | | **Women (n=193)** | | | | |
| --- | --- | --- | --- | --- | --- | --- | --- | --- | --- | --- | --- | --- | --- | --- | --- |
|  | **SCAA**  **(n=204)** | | **Non-SCAA**  **(n=206)** | |  | **SCAA**  **(n=152)** | | **Non-SCAA**  **(n=65)** | |  | **SCAA**  **(n=52)** | | **Non-SCAA**  **(n=141)** | |  |
|  | n/Mean | %/(SD) | n/Mean | %/(SD) | **SMD** | n/Mean | %/(SD) | n/Mean | %/(SD) | **SMD** | n/Mean | %/(SD) | n/Mean | %/(SD) | **SMD** |
| Number of teeth | 25.5 | (3.1) | 26.4 | (2.0) | -0.33 | 25.7 | (3.0) | 26.3 | (2.0) | -0.24 | 24.9 | (3.3) | 26.4 | (1.9) | -0.54 |
| Number of missing teeth | 2.1 | (3.1) | 1.1 | (1.8) | 0.37 | 1.9 | (3.0) | 1.3 | (1.9) | 0.23 | 2.5 | (3.2) | 1.1 | (1.7) | 0.57 |
| Number of implants | 0.3 | (1.0) | 0.1 | (0.4) | 0.20 | 0.2 | (1.0) | 0.2 | (0.6) | 0.02 | 0.4 | (0.9) | 0.1 | (0.3) | 0.44 |
| Number of replaced teeth | 0.7 | (1.8) | 0.4 | (1.0) | 0.23 | 0.6 | (1.7) | 0.5 | (1.0) | 0.10 | 1.0 | (2.2) | 0.3 | (0.9) | 0.42 |
| Number of lost teeth not replaced | 1.3 | (2.0) | 0.8 | (1.3) | 0.34 | 1.3 | (2.1) | 0.9 | (1.4) | 0.25 | 1.5 | (1.9) | 0.7 | (1.3) | 0.47 |
|  |  |  |  |  |  |  |  |  |  |  |  |  |  |  |  |
| Number of teeth with PPD ≥4 mm | 10.6 | (7.1) | 9.8 | (6.3) | 0.12 | 10.6 | (6.9) | 11.2 | (6.6) | -0.08 | 10.5 | (7.6) | 9.1 | (6.0) | 0.20 |
| Number of teeth with PPD ≥6 mm | 1.0 | (2.3) | 0.6 | (1.4) | 0.23 | 0.9 | (2.3) | 0.8 | (1.8) | 0.08 | 1.2 | (2.3) | 0.5 | (1.1) | 0.43 |
|  |  |  |  |  |  |  |  |  |  |  |  |  |  |  |  |
| Bleeding sites % |  |  |  |  |  |  |  |  |  |  |  |  |  |  |  |
| <10% | 9 | 4.4% | 6 | 2.9% | 0.09 | 6 | 3.9% | 0 | 0.0% | 0.31 | 3 | 5.8% | 6 | 4.3% | 0.07 |
| 10-29% | 54 | 26.5% | 60 | 29.1% |  | 37 | 24.3% | 13 | 20.0% |  | 17 | 32.7% | 47 | 33.3% |  |
| ≥30% | 141 | 69.1% | 140 | 68.0% |  | 109 | 71.7% | 52 | 80.0% |  | 32 | 61.5% | 88 | 62.4% |  |

Table S4: Self-reported symptoms reported separately (univariate analysis) and conjoined with Model 2 (see Table 3).

|  | **All (n=410)** | | | **Men (n=217)** | | | **Women (n=193)** | | |
| --- | --- | --- | --- | --- | --- | --- | --- | --- | --- |
|  | **Univariate** | **Model 2 + Q** | | **Univariate** | **Model 2 + Q** | | **Univariate** | **Model 2 + Q** | |
|  | *OR (95% CI)* | *OR (95% CI)* | *AUC* | *OR (95% CI)* | *OR (95% CI)* | *AUC* | *OR (95% CI)* | *OR (95% CI)* | *AUC* |
| **Q1 Swollen gums** | 0.93 (0.59 - 1.49) | 1.31 (0.76 - 2.27) | 0.80 | 0.82 (0.40 - 1.68) | 1.20 (0.55 - 2.63) | 0.70 | 1.43 (0.70 - 2.90) | 1.52 (0.67 - 3.45) | 0.78 |
|  | *N= 396* | | | *N=209* | | | *N=187* | | |
| **Q2 Sore gums** | 0.61 (0.41 - 0.90)* | 0.65 (0.41 - 1.03) | 0.80 | 0.38 (0.20 - 0.69) ** | 0.43 (0.23 - 0.82) * | 0.73 | 0.94 (0.49 - 1.79) | 0.97 (0.46 - 2.06) | 0.77 |
|  | *N=404* | | | *N=214* | | | *N=190* | | |
| **Q3 Receding gums** | 0.49 (0.31 - 0.76)** | 0.73 (0.43 - 1.24) | 0.81 | 0.58 (0.30 - 1.14) | 0.66 (0.32 - 1.36) | 0.73 | 0.69 (0.34 - 1.39) | 0.68 (0.30 - 1.57) | 0.77 |
|  | *N=362* | | | *N=194* | | | *N=168* | | |
| **Q4 Lose teeth** | 1.43 (0.56 - 3.64) | 1.17 (0.37 - 3.63) | 0.80 | 1.17 (0.30 - 4.57) | 1.43 (0.31 - 6.67) | 0.71 | 1.71 (0.40 - 7.44) | 1.26 (0.22 - 7.40) | 0.78 |
|  | *N=403* | | | *N=214* | | | *N=189* | | |
| **Q5 Drifting teeth** | 1.06 (0.51 - 2.20) | 1.32 (0.55 - 3.13) | 0.80 | 0.86 (0.28 - 2.61) | 1.06 (0.31 - 3.51) | 0.70 | 1.67 (0.57 - 4.88) | 1.98 (0.56 - 7.01) | 0.79 |
|  | *N=391* | | | *N=212* | | | *N=179* | | |
| **Q6 Bad breath** | 0.86 (0.52 - 1.44) | 1.10 (0.60 - 2.03) | 0.81 | 0.72 (0.34 - 1.51) | 0.91 (0.42 - 2.01) | 0.70 | 1.09 (0.48 - 2.48) | 1.40 (0.50 - 3.93) | 0.80 |
|  | *N=355* | | | *N=189* | | | *N=166* | | |
| **Q7 Toothache** | 0.70 (0.42 - 1.17) | 0.73 (0.40 - 1.34) | 0.80 | 0.87 (0.40 - 1.92) | (0.43 - 2.33) | 0.70 | 0.64 (0.27 - 1.50) | 0.52 (0.19 - 1.41) | 0.78 |
|  | *N=406* | | | *N=214* | | | *N=192* | | |
|  | | | | | | | | | |

Table S5: Self-reported oral hygiene habits of study participants.

|  | **SCAA**  **(n=204)** | **Non-SCAA**  **(n=206)** |
| --- | --- | --- |
| **Bleeding index** | 0.418 (0.196) | 0.396 (0.175) |
| ***OH habits*** |  |  |
| **Toothbrush type** |  |  |
| Manual | 82 (40.2%) | 72 (35.0%) |
| Electrical | 122 (59.8%) | 134 (65.0%) |
| **Frequency brushing** |  |  |
| 1/day | 29 (14.2%) | 8 (3.9%) |
| 2/day | 167 (81.9%) | 194 (94.2%) |
| 3/day | 8 (3.9%) | 4 (1.9%) |
| **Approximal cleaning** |  |  |
| None | 27 (13.2%) | 19 (9.2%) |
| Flossing | 54 (26.5%) | 77 (37.4%) |
| Interdental brush | 49 (24.0%) | 47 (22.8%) |
| Flossing & Interdental brush | 33 (16.2%) | 31 (15.0%) |
| Sticks/plastic pics | 41 (20.1%) | 32 (15.5%) |

Table S6: Self-reported dental treatment over the past 12 months.

|  | **SCAA**  **(n=204)** | **Non-SCAA**  **(n=206)** |
| --- | --- | --- |
| Examination | 164 (80.8%) | 165 (80.5%) |
| Dental filling | 84 (41.4%) | 63 (30.7%) |
| Scaling | 147 (72.4%) | 145 (70.7%) |
| Root canal treatment | 8 (3.9%) | 16 (7.8%) |
| Dental crown | 33 (16.3%) | 23 (11.2%) |
| Tooth extraction | 15 (7.4%) | 14 (6.8%) |
| Dental implant | 8 (3.9%) | 2 (1.0%) |

Figure S1: Flowchart

#### Figure S2: Descriptive overlook of number of missing teeth and prosthodontic replacements in SCAA and non-SCAA groups, stratified by gender. Categories assessed include number of missing teeth, number of replaced/not replaced missing teeth and number of implants.


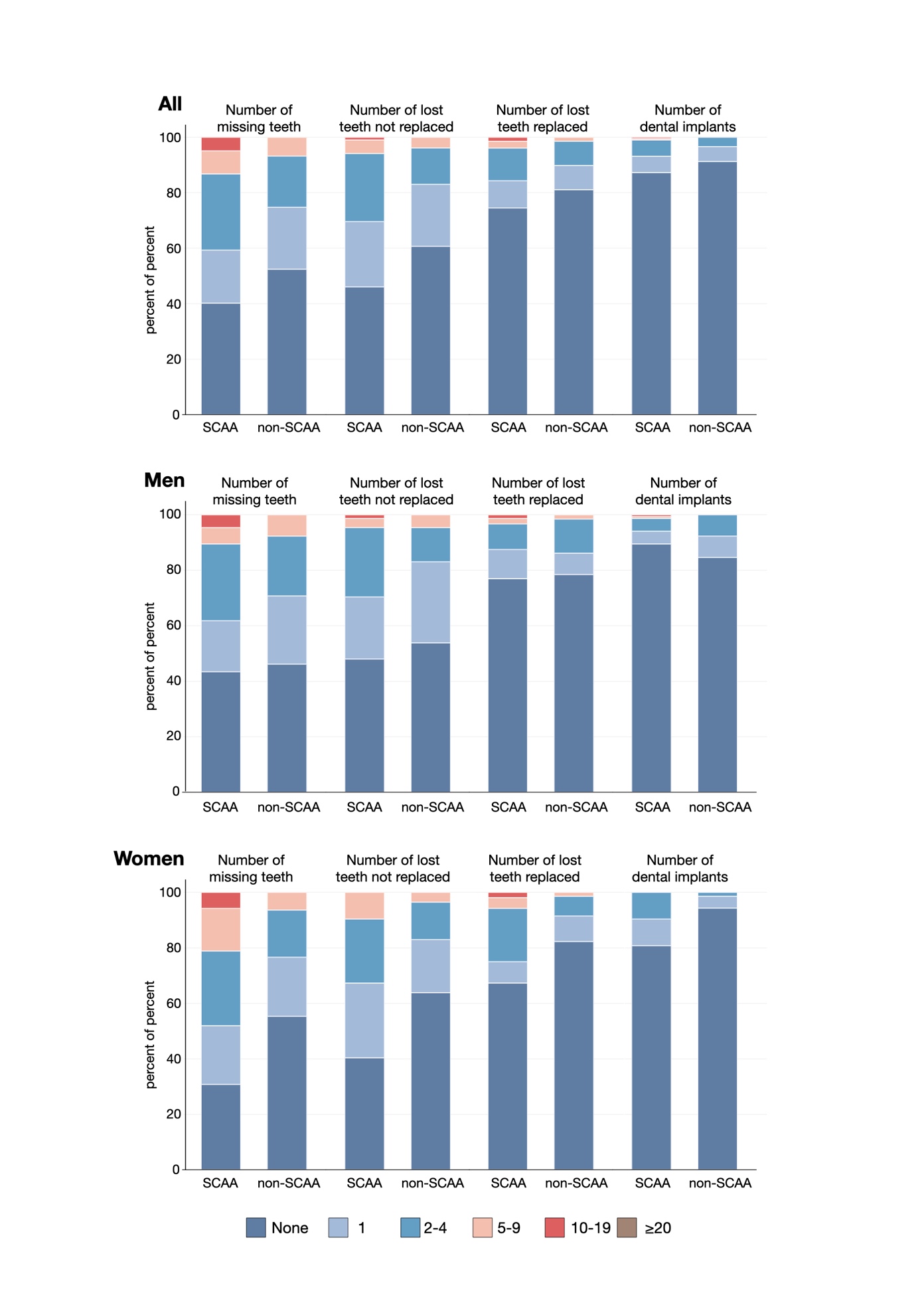


Figure S3: Extended logistic regression model (dependent parameter: SCAA) corresponding to Model 1 (Table 3) including personal/medical information. Area over the receiver operating characteristics curve (AUC) for the accuracy in the detection of SCAA.

| \| n=410 \| *Univariate* \| \| *Fully adjusted model* \| \| \| --- \| --- \| --- \| --- \| --- \| \|  \| OR (95% CI) \| *p* \| OR (95% CI) \| *p* \| \| Sex (ref. women) \| 6.34 (4.12 - 9.75) \| ** \| 7.82 (4.63 - 13.21) \| ** \| \| Age \| 1.12 (1.07 - 1.18) \| ** \| 1.11 (1.05 - 1.18) \| ** \| \| History of smoking: Pack-years \| 1.05 (1.02 - 1.08) \| ** \| 1.05 (1.02 - 1.09) \| ** \| \|  \|  \|  \|  \|  \| \| Dyslipidemia \| 7.03 (3.97 - 12.43) \| ** \| 3.87 (1.92 - 7.78) \| ** \| \| Hypertension \| 5.21 (3.34 - 8.13) \| ** \| 2.89 (1.61 - 5.16) \| ** \| \| BMI \| 1.07 (1.02 - 1.13) \| ** \| 0.98 (0.91 - 1.05) \|  \| \|  \|  \|  \|  \|  \| \| Frequency having an alcoholic drink, last year (ref: never) \|  \|  \|  \|  \| \| Once/month or less \| 0.73 (0.28 - 1.90) \|  \| 0.38 (0.12 - 1.22) \|  \| \| 2-4/month \| 0.81 (0.34 - 1.89) \|  \| 0.43 (0.16 - 1.20) \|  \| \| 2-3/week \| 0.68 (0.20 - 1.59) \|  \| 0.34 (0.12 - 0.95) \| * \| \| 4/week or more \| 1.15 (0.40 - 3.31) \|  \| 0.32 (0.09 - 1.18) \|  \| \|  \|  \|  \|  \|  \| \| Highest completed level of education (ref: not completed elementary school) \|  \|  \|  \|  \| \| Elementary school \| 1.86 (0.15 - 23.00) \|  \| 1.73 (0.11 - 27.53) \|  \| \| High school \| 2.30 (0.21 - 25.89) \|  \| 3.03 (0.22 - 41.43) \|  \| \| University \| 1.78 (0.16 - 19.97) \|  \| 2.49 (0.18 - 34.10) \|  \| \| ** p<0.01, * p<0.05  *BMI: Body Mass Index. DFT: Decayed and Filled Teeth. SCAA: Subclinical Coronary Artery Atherosclerosis* \| \| \| \| \| |  |
| --- | --- | --- | --- | --- | --- | --- | --- | --- | --- | --- | --- | --- | --- | --- | --- | --- | --- | --- | --- | --- | --- | --- | --- | --- | --- | --- | --- | --- | --- | --- | --- | --- | --- | --- | --- | --- | --- | --- | --- | --- | --- | --- | --- | --- | --- | --- | --- | --- | --- | --- | --- | --- | --- | --- | --- | --- | --- | --- | --- | --- | --- | --- | --- | --- | --- | --- | --- | --- | --- | --- | --- | --- | --- | --- | --- | --- | --- | --- | --- | --- | --- | --- | --- | --- | --- | --- | --- | --- | --- | --- | --- | --- | --- | --- | --- | --- | --- | --- | --- | --- | --- | --- | --- | --- | --- | --- |
